# Outcomes after elevation of serratus anterior fascia flap versus serratus muscle flap in direct-to-implant breast reconstruction following mastectomy: a prospective study

**DOI:** 10.61622/rbgo/2024AO13

**Published:** 2024-03-15

**Authors:** Lilian de Sá Paz Ramos, Jorge Villanova Biazús

**Affiliations:** 1 Federal University of Rio Grande do Sul Porto Alegre RS Brazil Federal University of Rio Grande do Sul, Porto Alegre, RS, Brazil.

**Keywords:** Breast reconstruction, Breast implants, Breast neoplasms, Mastectomy, Postoperative period, Pain, Fascia

## Abstract

**Objective:**

The purpose of this study was to compare postoperative pain between SF flap and serratus anterior muscle (SM) in direct-to-implant breast reconstruction.

**Methods:**

This is a prospective cohort study that included 53 women diagnosed with breast cancer who underwent mastectomy and one-stage implant-based breast reconstruction from January 2020 to March 2021. Twenty-nine patients (54.7%) had SF elevation, and 24 patients (45.3%) underwent SM elevation. We evaluated patient-reported early postoperative pain on the first day after surgery. Also, it was reported that all surgical complications in the first month and patient reported outcomes (PROs) were measured with the BRECON 23 questionnaire.

**Results:**

The serratus fascia group used implants with larger volumes, 407.6 ± 98.9 cc (p < 0.01). There was no significant difference between the fascial and muscular groups regarding the postoperative pain score reported by the patients (2 versus 3; p = 0.30). Also, there was no difference between the groups regarding early surgical complications and PROs after breast reconstruction.

**Conclusion:**

The use of SF seems to cause less morbidity, which makes the technique an alternative to be considered in breast reconstruction. Although there was no statistical difference in postoperative pain scores between the fascia and serratus muscle groups.

## Introduction

One of the benefits of direct-to-implant breast reconstruction is to allow rapid recovery of the breast, preserving self-image, which is fundamental for self-esteem and quality of life, as well as contributing to a reduction in the number of surgical procedures and hospital visits.^(1,2)^ The positioning of the implant, below the pectoralis major muscle, protects the integrity of the implant, reducing the visibility and palpability of the implant, besides reducing the occurrence of rippling.^(3,4)^ The pectoralis major muscle, in the subpectoral technique, covers about 2/3 of the implant. In order to enable a full coverage of the inferolateral portion of the prosthesis, the options are to use the serratus anterior muscle (SM), serratus anterior fascia (SF), de-epithelialized dermal flaps or the use of synthetic meshes and acellular-dermal matrix.^(5)^

The SF flap in breast reconstruction can be a safe and effective option for recreating the lateral profile of the breast and preventing lateralization of the implant. The advantage of this flap is that it is an autologous and well-vascularized tissue, which makes costal disinsertion of the serratus anterior muscle unnecessary, thus causing minimal impact on donor site morbidity and functionality.^(6,7)^ Despite potential advantages of less operative pain with the use of the SF flap, analytical studies evaluating the surgical outcomes of using this fascia in breast reconstruction, are scarce in the literature.^(1)^ This is a prospective cohort study to analyze the use of SF integrated one-stage implant-based breast reconstruction in women diagnosed with breast cancer by assessing pain postoperative outcome as primary endpoint.

## Methods

The present work was performed upon approval by the Ethics and Scientific Committee of the Aristides Maltez Hospital, approval number 3.722.354. Informed consent was obtained from all patients before being enrolled in the study.

This is a prospective cohort study that analyzed women diagnosed with breast cancer who underwent mastectomy and direct-to-implant breast reconstruction from January 2020 to March 2021, with a follow-up period of 3 months. Patients were distinguished into two groups according to the elevation of the SF or SM for the creation of the prosthetic pocket. All surgical procedures were performed by three breast surgeons with more than ten years of practical experience in oncologic surgery. The distinction between the participants who had fascia or serratus anterior muscle elevation was based on the surgical description. All information was obtained from hospital chart records.

Inclusion criteria for the study were women diagnosed with intraductal and invasive breast carcinoma, clinical stage 0 to III undergoing skin and/or areola sparing mastectomy and single-stage breast reconstructive surgery with direct-to-implant by the subpectoral technique and SF or SM elevation for complete inferolateral coverage of the prosthesis. Patients with a diagnosis of inflammatory breast cancer, tumor recurrence, distant metastases, as well as the use of alloplastic materials in the reconstructive technique, such as synthetic or biological meshes, for prosthesis coverage or partial prosthetic pocket performance were excluded.

The clinical variables analyzed were age, menopausal status, body mass index (BMI), smoking, comorbidities, histological type, tumor grade, biological subtype, clinical staging, neoadjuvant chemotherapy, type of mastectomy and axillary approach, and prosthesis volume. The outcomes investigated were postoperative pain on the first day after surgery and early surgical complications. The postoperative pain reported by the patient was measured with the visual numerical scale on the first postoperative day, which establishes a score from 0 (no pain) to 10 (maximum pain). Major surgical complications were those requiring rehospitalization or reoperation, and implant loss. Minor surgical complications were defined as postoperative courses without the need of surgical interventions and resolved on an outpatient basis. The complications recorded were surgical site infection, seroma, hematoma, epidermolysis, skin flap necrosis and/or partial or total areola papillary complex, wound dehiscence, and implant loss. The criteria for defining surgical site infection were administration of antibiotics beyond the surgeon's standard perioperative period with or without the presence of localized clinical signs of infection (erythema, pain, swelling, or hyperthermia). Hematoma and seroma were considered when puncture was needed for drainage of the liquid collection.

The instrument used to measure the PROs was the BRECON 23 questionnaire developed by the European Organization for Research and Treatment of Cancer. The BRECON 23 integrates five multi-item scales to assess body image, sexual functioning, systemic therapy side effects, breast symptoms, and arm symptoms. For each subscale of the questionnaire was calculated a score from 0 to 100, in which a high score represented an elevated level of functionality or high level of symptoms or problems.^(8)^ In this study, the BRECON 23 was completed at a median of 106 days after immediate breast reconstruction following mastectomy. Subscales were scored as per published scoring system.

All the participants in this study had skin sparing or nipple-areola sparing mastectomy and the axillary approach, which consisted of sentinel lymph node biopsy or axillary lymphadenectomy. The breast reconstructive technique used was subpectoral with elevation of the pectoralis major muscle, through dissection of the muscle fibers from ribs and sternal insertion, leaving its covering fascia (superficial pectoral fascia) intact down to the inframammary line fold to maintain the continuity of the pocket roof.

**Figure 1:**
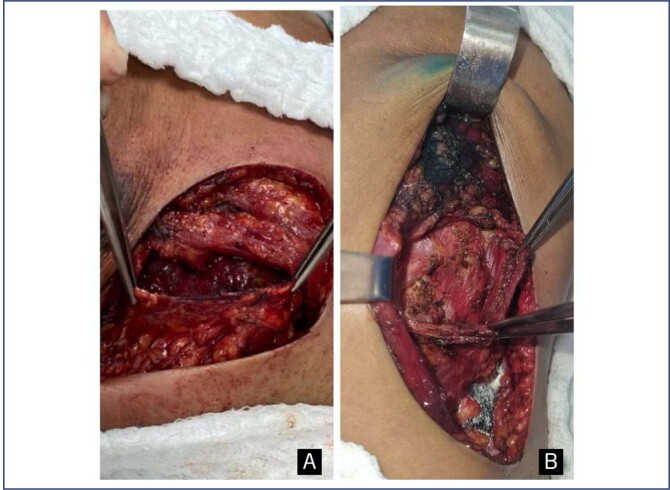
Intraoperative photograph of elevation the serratus anterior fascia and muscle for lateral coverage of breast implant. a - anterior serratus fascia flap; b - serratus anterior muscle flap

To inferolateral coverage of the border of the prosthesis, the fascia or serratus muscle flap was used. Laterally the fascia overlying the serratus anterior muscle was meticulously elevated off the serratus muscle in continuity with the lateral border of pectoralis major, preserving the integrity of the muscle in a group of patients or the fibers of the serratus anterior muscle flap was detached its costal insertions in another group of patients (Figure 1).

All patients had direct implantation of round textured silicone prosthesis positioned in the subpectoral plane and a complete coverage by suturing the lateral border of the pectoralis major muscle with the SM or SF flap, thus enabling complete coverage of the inferolateral implant. Tubular drains under vacuum suction were inserted into the prosthetic site and subcutaneous tissue, which was maintained until drainage was less than 50ml. No surgery was performed on the contralateral breasts for the purpose of symmetrization. The Statistical Package for Social Sciences (SPSS) program, version 18.0. was used for data analysis. The chi-square test was used to analyze the proportion and association of categorical variables. For quantitative variables, the Shapiro-Wilk normality test was applied. For variables with normal distribution, the independent t test was used, and for variables with non-normal distribution, the Mann-Whitney U test was used. The results were considered statistically significant for p-values less than 0.05. For the postoperative pain outcome, the sample size was calculated using the Sealed Envelope® Sample Size software, based on the study by Bordoni et al.,^(7)^ which found a lower pain scores reported by the patients in the subfascial group 5.85 (SD 0.87) versus 7.10 (SD 1.04) at 24h, p<0,05 .^(7)^ Considering a confidence interval of 95%, a significance level of 5% and possible losses of 20% of patients, a sample of 31 participants would be needed to find a difference of 5%.

## Results

A total of 53 women diagnosed with breast cancer who underwent mastectomy associated with immediate subpectoral reconstruction were included in the study, 29 participants had SF elevation and 24 participants, SM elevation. The mean age of the population was 42.6 ± 8.2 years, most were premenopausal 84.9% and 52.9% had a BMI over 24.9 Kg/m². Thirty-four percent had at least one of the following comorbidities: systemic arterial hypertension; diabetes; psychiatric disease; fibromyalgia; cardiac disease; thyroid disease and asthma. The demographic, clinical and pathological characteristics of both groups were similar (Table 1).

**Table 1:** Demographic, clinical, and pathological characteristics of the population

Characteristics		Total n = 53 n(%)	SF group n = 29 n(%)	SM group n = 24 n(%)	p-value
Age, year		42.6 ±*8.2*	43.7 ± 9.0	41.25 ± 7.1	0.29
Menopause status					0.27
	Pre-menopause	45(84.9)	23(79.3)	22(91.7)	
	Post-menopause	8(15.1)	6(20.7)	2(8.3)	
Smoking					0.61
	Not	44(83)	23(79.3)	21(87.5)	
	Ex-smoker	2(3.8)	2(6.9)	0(0)	
	Smoker	7(13.2)	4(13.8)	3(12.5)	
BMI, Kg/m²		25.8 ± 4.4	25.8 ± 3.7	25.8 ± 5.2	1.00
BMI classification					1.00
	Underweight	2(3.8)	1(3.4)	1(4.2)	
	Normal	23(43.4)	13(44.8)	10(41.7)	
	Overweight	18(34.0)	10(34.5)	8(33.3)	
	Obese	10(18.9)	5(17.2)	5(20.8)	
Comorbidities					0.70
	Not	35(66.0)	18(62.1)	17(70.8)	
	Yes	18(34.0)	11(37.9)	7(29.2)	
Neoadjuvant chemotherapy					0.80
	Not	31(58.5)	16(55.2)	15(62.5)	
	Yes	22(41.5)	13(44.8)	9(37.5)	
Grade, B&R					0.49
	1	7(13.5)	3(10.3)	4(17.4)	
	2	30(57.7)	16(55.2)	14(60.9)	
	3	15(28.8)	10(34.5)	5(21.7)	
Histology classification					0.13
	DCIS	4(7.5)	2(6.9)	2(8.3)	
	No special type	45(84.9)	25(86.2)	20(83.4)	
	Ductal	41(77.4)	25(86.2)	16(66.7)	
	Lobular	4(7.5)	0(0)	4(16.7)	
	Special types	4(7.5)	2(6.9)	2(8.3)	
Stage, n (%)					0.60
	0	4(7.5)	3(10.3)	1(4.2)	
	I	10(18.9)	5(17.2)	5(28.8)	
	II	31(58.5)	18(62.1)	13(54.2)	
	III	8(15.1)	3(10.3)	5(20.8)	
ER status*					1.00
	Positive	42(82.4)	24(82.8)	18(81.8)	
	Negative	9(17.6)	5(17.2)	4(18.2)	
PR status					0.55
	Positive	36(70.6)	19(65.5)	17(77.3)	
	Negative	15(29.4)	10(34.5)	5(22.7)	
HER2					0.64
	No overexpression	46(90.2)	27(93.1)	19(86.4)	
	Overexpression	5(9.8)	2(6.9)	3(13.6)	
Biological subtype					0.81
	Luminal A	20(39.2)	11(37.9)	9(40.2)	
	Luminal B	23(45.1)	14(48.3)	9(40.9)	
	Triple-negative	7(13.7)	4(13.8)	3(13.6)	
	HER2-enriched	1(2.0)	0(0)	1(4.5)	

SF, serratus fascia; MF - muscle serratus; BMI - body mass index; DCIS - ductal carcinoma in situ; ER - estrogen receptor; PR -progesterone receptor; HER2 - human epidermal growth factor receptor type 2;

* evaluated 51 participants

There was no difference between the SF and SM groups regarding the type of mastectomy, axillary approach, and laterality, therefore, the SF group used implants of larger volume, 407.6 ± 98.9 cc (p < 0.01). Twenty-seven patients (50,9%) had the nipple areolar complex preserved (Table 2).

**Table 2:** Surgery summary

Characteristic		Total n = 53 n(%)	SF group n = 29 n(%)	SM group n = 24 n(%)	p-value
Type of mastectomy, n (%)					0.60
	Skin-sparing	26(49.1)	13(44.8)	13(54.2)	
	Nipple-sparing	27(50.9)	16(55.2)	11(45.8)	
Axillary surgery, n (%)					0.25
	Sentinel lymph node biopsy	30(56.6)	19(65.5)	11(45.8)	
	Axillary lymphadenectomy	22(43.4)	10(34.5)	13(54.2)	
Laterality, n (%)					0.11
	Right	26(49.1)	11(37.9)	15(62.5)	
	Left	25(47.2)	16(55.2)	9(37.5)	
	Bilateral	2(3.8)	2(6.9)	0(0)	
Implant volume, cc		375.5 ± 97.3	407.6 ± 98.9	336.7 ± 81.3	0.007*

SF - serratus fascia; MF - muscle serratus; n - absolute number;

* p < 0.05

Pain on the first postoperative day was analyzed in 51 of the total 53 participants due to loss to follow-up of two participants in the fascia group. There was no difference in pain score reported by patients between fascial and muscle groups, median and 25th - 75th percentiles of pain intensity score was 2.0 (0 - 3.5) versus 3 (0 - 5.0) respectively, but without statistical significance (p = 0.3). All surgical complications that occurred in the first 30 days were minor surgical complications (Table 3). There were no major complications in the first month after surgery. Minor complications occurred in 8 patients (27.6%) in the SF group compared with 6 patients (25%) in the SM (*p* = 1.00).

**Table 3:** Summary of surgical complications

Complications	Total n = 53 n(%)	SF group n = 29 n(%)	SM group n = 24 n(%)	p-value
Total complications*	14(26.4)	8(27.6)	6(25.0)	1.00
Infection	4(7.5)	2(6.9)	2(8.3)	1.00
Hematoma	2(3.8)	2(6.9)	0(0)	0.50
Seroma	5(9.4)	3(10.3)	2(8.3)	1.00
Epidermolysis	8(15.1)	4(13.8)	4(16.7)	1.00
Flap necrosis	2(3.8)	2(6.9)	0(0)	0.50
Areola necrosis	2(3.8)	2(6.9)	0(0)	0.50
Wound dehiscence	1(1.9)	0(0)	1(4.2)	0.45

SF - serratus fascia; MF - muscle serratus; n - absolute number;

* participants with more than one complication were counted once

**Figure 2:**
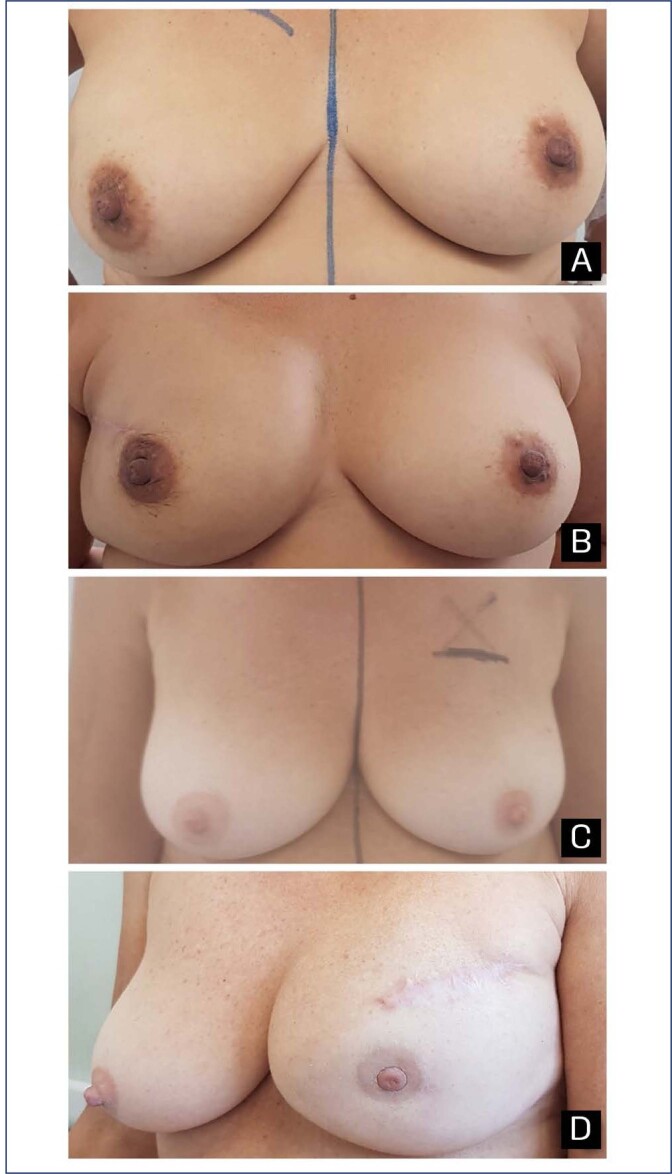
Aesthetic outcome: a preoperative photograph of a 45-year-old patient with right breast cancer, b postoperative photograph of right subpectoral direct-to-implant breast reconstruction with the use of serratus muscle integrated (535cc implant volume); c preoperative photograph of a 58-year-old patient with left breast cancer, d postoperative photograph of left subpectoral direct-to-implant breast reconstruction with the use of serratus fascia

BRECON 23 was completed by 26 of 29 patients (89.6%) in the serratus fascia group and by 22 of 24 (91.7%) in the serratus muscle group. When comparing the score by subscale of the SF and SM groups, there was no median difference in satisfaction with breast cosmetics (77.8 versus 83.3; p=0.81) and surgery side-effects (16.7 versus 33.3; p=0.21). All patients who had a nipple-sparing mastectomy considered that the preservation of the nipple helped a lot to accept the treatment or disease (Figure 2).

## Discussion

In 2010, the use of serratus fascia in breast reconstruction was first described by Saint-Cyr et al.,^(6)^ the authors concluded that the use of SF is a safe, effective, and inexpensive method for lateral coverage of the tissue expander and reconstruction of the lateral breast profile, promoting a good aesthetic result with minimal complications. In addition to avoiding the additional costs and inherent risks of using other alloplastic materials such as biological and synthetic meshes.^(6)^ Similarly, our study found that the use of SF in reconstruction with direct-to-implant is safe, there was no difference regarding the occurrence of surgical complications or reports of satisfaction with breast cosmetic compared to the classical technique of SM elevation.

Most studies on postoperative pain followed by breast reconstruction evaluate chronic pain more than acute pain. But patients who suffer with less control of postoperative pain report less satisfaction with surgery.^(9)^ Since the manifestation of post-surgical pain negatively influences quality of life, it is important that it be minimized.^(10)^ Previous studies have reported the potential advantage of using serratus fascia for presenting less functional impairment and pain associated with costal disinsertion of the serratus anterior muscle.^(6,11)^ However, only Bordoni et al.^(7)^ quantitatively evaluated the outcome of pain with the use of serratus fascia in breast reconstruction with direct implantation, in which he found lower patient-reported early postoperative pain scores at 24 hours and 5 days postoperatively with a statistically significant difference favorable to the fascia group compared to the muscle group (p < 0.05). Our results did not confirm this difference in pain reporting on the first postoperative day between the fascial and muscle groups (p = 0.30).

Besides, Seth et al.^(12)^ considered that the ideal patient to use the technique is one without comorbidities, history of radiotherapy or axillary dissection performance, and an average BMI.^(6)^ Still Chan et al.^(11)^ also found that SF is safe and versatile for performing subpectoral breast reconstruction with direct implantation and emphasized the importance of selecting women with small to medium breasts, in addition to meticulous execution of the procedure for the success of the technique. Also unlike these studies, we did not observe difference between the groups regarding the presence of comorbidities, performance of axillary dissection and BMI. These variables do not seem to interfere in fascial flap preparation. Therefore, our results suggest that these criteria should not be considered limiting when selecting patients to perform the technique with serratus anterior fascia, which diverges from the conclusions of previous studies that suggested an ideal patient profile for performing the fascia technique.

The serratus fascia group of patients used larger implants, a mean of 407.6 ± 98.9 cm³ (p < 0.01). A similar result was found in the study by Seth et al.,^(12)^ in which they reported that patients with serratus fascia elevation obtained a larger intraoperative tissue expander fill volume compared to serratus muscle elevation (p < 0.01). The authors showed that fascial tissue is thinner and more distensible than muscle tissue, thus creating a larger potential space for expansion.

According to the measurement of patient reports using the BRECON 23, both groups expressed equivalent satisfaction with breast reconstruction. Our results demonstrate that the choice between fascia or serratus anterior muscle does not interfere with patient-reported outcomes satisfaction with the outcome of breast reconstruction.

In this prospective study, despite the limitations of not being a randomized clinical trial and having a short period of observation that limits inferences only from the early results of the surgery, we observed that the use of SF integrated with the making of the subpectoral implant pocket is a feasible technique that maintains a surgical complication rate and patient satisfaction with a reconstructive result similar to the classical technique. However, the results of the fascial group point to a propensity to less functional impact and pain in the postoperative period, which would make preferable the surgeon's choice of using serratus fascia to cover the inferolateral portion of the prosthesis in relation to the elevation of the serratus anterior muscle, thus favoring less morbidity for the patients.

## Conclusion

This prospective study evaluated the early postoperative pain of patients undergoing immediate subpectoral breast reconstruction with direct prosthesis inclusion, comparing the use of SF and SM for coverage of the inferolateral part of the implant. There was no statistical difference between the fascial and muscle groups, the pain scores pain scores reported by patients. However, the results point to a lower propensity for functional impact and morbidity with the use of SF, which would make this technique preferable for coverage of the inferolateral portion of the prosthesis. Also, according to our results, the technique can be applied in patients without selection restrictions involving BMI, presence of comorbidities or performance of axillary dissection. Future studies with larger numbers of patients and longer observation time will be necessary to confirm the benefit of this technique.
